# Internal Quality and Job Satisfaction in Health Care Services

**DOI:** 10.3390/ijerph19031496

**Published:** 2022-01-28

**Authors:** Aspasia Goula, Theodoros Rizopoulos, Maria-Aggeliki Stamouli, Martha Kelesi, Evridiki Kaba, Sotirios Soulis

**Affiliations:** 1 Department of Business Administration, School of Administrative, Economics and Social Sciences, University of West Attica, 12243 Athens, Greece; thodorizopoulos@gmail.com (T.R.); mstamouli@yahoo.com (M.-A.S.); soulsot@uniwa.gr (S.S.); 2 Department of Nursing, School of Health and Care Sciences, University of West Attica, 12243 Athens, Greece; mkel@uniwa.gr (M.K.); ekaba@uniwa.gr (E.K.)

**Keywords:** internal service quality, internal marketing, job satisfaction, internal customers’ perception, health management

## Abstract

(1) Background: The main purpose of this study was to evaluate the health services’ internal quality level in Greek public hospitals and to investigate whether there is a relation between internal quality and health care professionals’ job satisfaction. (2) Material and Methods: A cross-sectional study was conducted in six public hospitals (four general and two specialized hospitals). The following tools were used to collect data: (a) the SERVQUAL questionnaire, which is designed to measure service quality through five dimensions, and (b) the Job Satisfaction Survey (JSS) questionnaire, which is designed to measure employees’ job satisfaction. Convenience sampling was used as a sampling technique. (3) Results: The level of internal quality service was found to be low as regards the dimensions of: reliability, assurance, responsiveness, and empathy, while the “tangibles” dimension was the only one which was assessed as having a satisfactory internal quality level. Τhe results also revealed a positive correlation between the job satisfaction subscales and the quality dimensions. Regarding demographic characteristics and their effect on employees’ perceptions of internal quality dimensions, the study found that the gender and the educational factor had no effect while younger employees have a more positive perception of the quality of responsiveness dimension in the health organizations where they work. In terms of profession, administrative staff had a lower perception of the quality of tangibles dimension than doctors and nurses. Concerning years of experience, the results indicated that employees with more previous working experience had a worse perception of the quality of reliability, responsiveness, and assurance dimensions. (4) Conclusions: According to the results, establishing a sense of trust and understanding between management and health professionals through effective communication, transparent evaluation, and reward is critical to developing, enhancing, and promoting an internal quality culture in a hospital setting.

## 1. Introduction

In today’s labor market environment, service quality is regarded as one of the most important factors in determining an organization’s success [1]. Consequently, organizations, in order to acquire highly satisfied and loyal customers [2], need to provide services dependably and accurately [3] that meet or exceed customers’ expectations [4].

The concept of “customer-focused quality” has been debated for a long time in the scientific field of total quality management [5]. Until the 1980s, the framework quality-shaping idea was solely determined by how the external customers evaluate it.

After the decade of the 1980s, the internal marketing and internal quality philosophy, as well as its impact on service quality, became a new topic in service provision, and since then, an increasing number of researchers have recognized its critical role in generating results and they have explained the relationship between internal marketing and service quality [6,7,8].

Berry et al. [9] were the first who introduced the term “internal marketing”. According to them, internal marketing refers to all the activities that a company must undertake in order to develop, train, and encourage its employees to improve the quality of services provided to its customers [10]. The researchers described internal marketing as “viewing employees as internal customers, viewing jobs as internal products that satisfy the needs and wants of internal customers while addressing the needs of external customers” [3]. Additionally, Gronroos emphasized that employees served as a significant element of the entire product or service given by an organization and he described employees at all levels of the organization as internal customers, who should be trained as marketers with special abilities in order to maintain long-term customer relationships [11,12,13]. This approach is viewed by marketing, business management, and organizational behavior researchers as a vehicle for implementing the organization’s strategies [14].

The internal marketing concept is based on the idea that organizations that attempt to serve external customers must initially serve the needs of their internal customers [15,16] and employees must be both motivated and customer-oriented in order to achieve service excellence [17]. As a result, employees should be regarded as internal customers and the organization should treat employees in a way that promotes job satisfaction and encourages them to be more productive in order to enhance organizational effectiveness, organizational success, service quality, customer satisfaction, productivity, and loyalty [18,19,20,21,22].

Internal customers suggest that every employee is both a seller and a buyer of services to other employees within the same organization [22]. As a result, individual divisions must regard themselves as both customers and suppliers since they receive a service or a product as input from a different department/division (their supplier), they add value to it, and afterwards they transmit the final product or the service as output to another department/division (their customer) [3]. As a result, before the product or service reaches external clients, an internal service chain is established [23]. An organization, from this perspective, is made up of a series of individuals and functional units linked together with the goal of meeting the needs of its external consumers [24,25]. The value of service that is provided to external customers is frequently determined by the value of service which is provided to internal customers [26]. An organization cannot provide the quality service that is promised to its external customers (patients) without the active involvement of all the internal customers (employees). Employees cannot deliver exceptional service to their customers unless they receive excellent service from other employees with whom they engage to provide the service. The quality of service delivered to the organization’s personnel frequently determines how well the external consumer is serviced [27].

Some researchers have argued that failing to satisfy the requirements of a participant in the internal quality chain creates problems at another stage of the chain [28]. When internal customers (the company’s employees) are satisfied, they are more willing to provide high-quality service to their customers (the external customers) [29]. Thus, each one of these interactions contributes to the overall satisfaction of the external customers and their willingness to continue using the organization’s services [30,31]. The internal customers are the people who benefit from the services they use. They are better informed and educated about the provided services than external clients [32].

An organization’s internal quality processes result in satisfied and loyal employees, [33,34] who, in turn, provide superior services to the firm’s external consumers [35]. Employees’ satisfaction is a concept that is studied in organizational sciences and refers to the employees’ feelings about their job and how they react to them [36]. However, it is only recently that academics have recognized the importance of the relationships between internal customers, service quality, and job satisfaction [1] and while the need for internal marketing is recognized, unfortunately, only a few organizations put internal marketing into practice [37].

Regarding health care service quality, most of the studies were focused on the external customers (patients), and less on how health care employees evaluate the delivery of health care quality. However, health care is a high-labor-intensive business that requires regular interactions between health care employees and customers–patients. Consequently, employees’ evaluation about internal quality and satisfaction is essential in order to provide high-quality services [38]. According to many studies, patients were more satisfied in health care organizations where employees were sensitive to internal marketing in order to provide high-quality services to patients [10,39,40]. Several recent studies have also shown that employees’ satisfaction is connected to customer satisfaction [41] and to quality of patient care [42,43,44,45,46,47,48].

In Greece, few of the internal quality studies have focused on health care service, particularly in public hospitals. Moreover, there is a gap in the literature examining the internal quality and job satisfaction in public health care services after the 2008–2018 period in which Greece was under an economic crisis. In this period, the public health system was underfunded [49], there was a reduction in the number of employees, drastic cuts in wages, and the abolition of the thirteenth monthly wage, due to the crisis [50].

The main purpose of this study was to evaluate the health services’ internal quality level in Greek public hospitals in the region of Attica. An additional objective was to investigate if there is a relation between internal quality and job satisfaction and also to explore if there are differences between the employees’ demographic characteristics and their perceptions regarding the level of internal quality services.

Therefore, the research questions that arise, about Greek hospitals, are the following:

a. What is the level of health services’ internal quality in Greek public hospitals?
b. Is there any relation between internal quality and job satisfaction?
c. Do employees with different demographic characteristics have different perceptions as regards the level of internal service quality?

## 2. Materials and Methods

### 2.1. Participants and Procedure

The survey was carried out in the region of Attica, Greece. According to OECD (Organization of Economic Cooperation and Development), in the capital of the country, the number of in-patient beds is three times higher than in other regions of the country. The same applies for human resources and available medical equipment [51]. The Attica region has 27 public hospitals that provided health services to 689,077 patients in 2020. Our survey was carried out in six of them that provided health care services to 269,156 patients (39 percent of the whole access population). Secondary care and tertiary care, as well as advanced primary care, are all offered by the specific hospitals. The main criteria for selecting these hospitals were: (a) the large number of patients frequently treated and accommodated in these hospitals; and (b) the large number of health care professionals who were working in these hospitals [52].

This study used a cross-sectional research design and convenience sampling as a sampling technique. This was a non-probability sampling method where the sample was taken from a group of health professionals that were easy to be contacted or reached (i.e., those who were more willing to participate in the survey). In total, 441 responded to this, representing a response rate of 75%.

The participants were adults (over 18 years), health care professionals belonging to medical, nursing, administrative, and technical departments. The research was conducted from 15th of May 2021 to 15th of September 2021.

All participants signed a written consent form before completing the survey. The researcher guaranteed the anonymity and confidentiality of all data collected. All subjects had the right to refuse or discontinue their participation in the study, according to the ethical standards of the Helsinki Declaration.

### 2.2. Research Instrument

The instrument used for data collection was the Greek version of: (a) the SERVQUAL Quality Questionnaire [53]; and (b) six questions from the Job Satisfaction Tool (JSS), which had been validated to measure internal quality for Greek health settings by Pantouvakis and Mpogiatzidis [54] and by Goula et al. [55].

SERVQUAL consists of 22 questions «that make up the five quality dimensions. The dimensions are the following: (i) Tangibles (4 items); (ii) Reliability (5 items); (iii) Responsiveness (4 items); (iv) Assurance (4 items); (v) Empathy (5 items). All the questions were ranked on a 7-point Likert scale, ranging from 1-totally disagrees to 7-totally agree, which means that higher scores show higher expectations and better evaluation of the received services. Studies, investigating internal service’s quality in general and in hospital environment particularly, concluded that SERVQUAL can be used to measure internal service’s quality» [11,24,34,54,55,56,57,58,59].

The six questions of JSS used for the study were: (i) satisfaction from earnings, (ii) satisfaction from opportunities to develop capabilities and initiatives, (iii) satisfaction from operating working conditions, (iv) satisfaction from contingent rewards, (v) satisfaction from colleagues and other hospital employees, and (vi) overall job satisfaction. All the questions were ranked on a 7-point Likert scale, ranging from 1-totally disagree to 7-totally agree.

The questionnaire had one more section regarding the demographic characteristics of the respondents (5 items).

To assess the questionnaire’s reliability, the Cronbach’s alpha coefficient was calculated separately for each quality dimension as well as for all the questions as a whole. In terms of job satisfaction subscales, the coefficient was calculated for the entire set of questions. As shown in the table below (Table 1), the coefficient values ranged from 0.795 to 0.964, and are considered to range from acceptable to excellent.

### 2.3. Statistical Analysis

**Figure 1:**
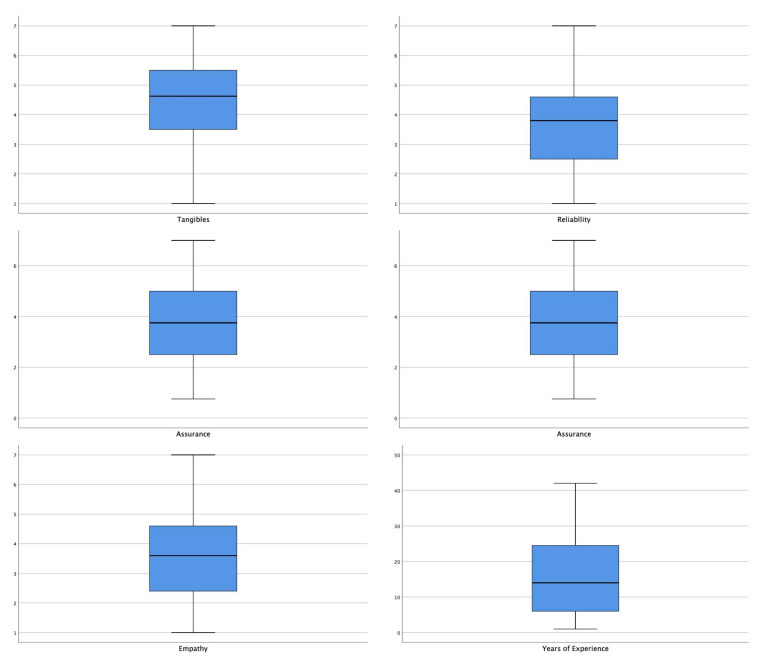
The boxplots of Years of Experience and the Quality dimensions.

For the statistical analysis, the SPSS 26 (IBM, Athens, Greece), was used. The quality dimensions were calculated as mean values of the questions that compose each one of them. Normality tests for the five quality dimensions as well as for “previous experience” revealed a statistically significant deviation from normality (Table 2). The respective boxplots (Figure 1), however, revealed no outliers or extreme skewness for any of those characteristics. As a result, the one-sample t-test was used to assess the statistically significant differences between the quality dimensions and the neutral value (neutral value = 4), while the independent samples *t*-test was used to determine the statistically significant differences of the internal quality level between the two independent groups of a dichotomous variable (i.e., gender).

Moreover, the one-way ANOVA test or the Brown–Forsythe test with post hoc analysis using Tukey HSD test and Games–Howell test (in cases where the one-way ANOVA or the Brown–Forsythe test was statistically significant), were employed to assess the statistically significant differences of the internal quality level among more than two groups. Finally, the non-parametric Spearman’s Rho correlation coefficient was used to assess the possible correlation between “Years of Experience” and the dimensions of quality, as well as between the Job Satisfaction subscales and the dimensions of quality. The level of statistical significance was set to α = 0.05 [60,61].

In the above boxplot (the boxplots are also called box and whisker plots), the horizontal lines within each box represent the median, the lower end of each box is the 1st quartile, the upper end of each box is the 3rd quartile, the boundary of the lowest whisker is the minimum value of the dataset, and the boundary of the highest whisker is the maximum value of the data set.

## 3. Results

### 3.1. Descriptive Analysis of the Sample

The questionnaires were distributed to 441 health professionals who were chosen using the convenience sampling method. Regarding the sampling frame, 72.6% of the participants were female and 27.4% were male. Most of the professionals in the survey were nurses (41.3%), 24.4% were administrative staff, 16.8% were doctors, and the remaining 17.9% were of other health professional specialties. In terms of age distribution, most of the participants (34.2%) belonged to the age group of (31–40) and 32.7% to the age group of (41–59). Finally, as regards their educational level, most of them (32.0%) were graduates of technological institutes, while their average professional experience was 15.88 ± 10.87 years, with a median value of 14 years (Table 3).

### 3.2. Overview of the Internal Quality Dimensions and Job Satisfaction Subscales

Table 4 shows the SERVQUAL questions that received the highest ratings from the participants in the survey, indicating that these are the questions that represent the highest-quality hospital services. Three out of five questions came under the Tangibles dimension, one under the Assurance dimension, and the last one under the Empathy dimension. Therefore, the participants seemed to have a very favorable impression of the staff’s physical appearance, which they described as “decent” and “professional”, of the equipment in the departments where they work, as well as of the attitude and the professionalism of the administrative employees.

Table 5, on the other hand, shows the five questions with the lowest ratings. Three out of these five questions fall under the dimension of Empathy, one under the dimension of Responsiveness, and the last one under the dimension of Assurance. This table clearly shows that employees did not believe that hospital managers give them individual attention, and they did not understand the needs and requirements of the department in which each employee works. Furthermore, the employees who participated in this research did not believe that management staff always provides services on time as promised, and they did not believe that managers inspire confidence in them.

Table 6, which follows, shows an overview of the level of selected job satisfaction subscales as reported by survey participants. It is clear from the table that the subscales with the highest scores are the following: (a) satisfaction from colleagues and other hospital employees (mean = 4.55), (b) overall job satisfaction (mean = 3.88), and (c) satisfaction resulting from contingent rewards for good work (mean = 3.14), while the subscales with the lowest scores are as follows: (a) satisfaction with the opportunities to develop capabilities and initiatives (mean = 3.10), (b) salary satisfaction (mean = 3.06), and, finally, (c) satisfaction with the operating working conditions (mean = 2.99). Based on the aforementioned, it appears that the employees who participated in this research are more satisfied with their interaction with their colleagues, with practicing their profession as a whole, and with people acknowledging their efforts at work, but they do not express the same level of satisfaction with the opportunities and possibilities for improving their skills, as well as with their earnings and the operating working conditions.

### 3.3. Assessing the Level of Internal Quality

In terms of the employees’ perceptions of the five quality dimensions level, the statistical analysis using the one-sample *t*-test revealed that all of the dimensions were statistically significantly different from the neutral value (neutral value = 4, which shows neither agreement nor disagreement). Specifically, all the dimensions, except for the “Tangibles” one, showed statistically significant lower mean value (Table 7). This finding suggests that, with the exception of the Tangibles dimension, the employees who participated in the study evaluated the quality of services in the hospitals where they work as non-satisfactory (Table 7).

### 3.4. Impact of Sociodemographic Factors on Internal Quality Dimensions

#### 3.4.1. The Gender Factor

In order to analyze the impact of the “Gender” factor on the internal quality dimensions, the statistical analysis with the one sample *t*-test revealed that the test was not significant for all the quality dimensions (tTAN = 0.157, *p* = 0.875, tREL = 0.657, *p* = 0.881, tRES = −0.204, *p* = 0.511, tASR = −0.022, *p* = 0.838, tEMP = −0.09, *p* = 0.928). This result indicates that the gender factor did not seem to affect employees’ perceptions regarding the internal quality of the hospital where they work (Table 3).

#### 3.4.2. The Age Groups Factor

As regards the “Age Groups” factor, the ANOVA test (or the Brown–Forsythe when Levene’s test for homogeneity of variance was statistically significant) showed statistically significant differences among the age groups only for the responsiveness dimension (B-FTAN = 0.565, *p* = 0.638, FREL = 2.484, *p* = 0.06, FRES = 2.995, *p* = 0.031, FASR = 2.186, *p* = 0.089, FEMP = 0.878, *p* = 0.453) (Table 3). The post hoc analysis which followed with the use of Tukey test (since Levene’s test for homogeneity of variance was not statistically significant, Levene’s (3, 437) = 1.54, *p* = 0.205)), showed that statistically significant differences exist only for the pair (≤30) (mean = 4.28, standard deviation = 1.42) vs. (≥51), (mean = 3.55, standard deviation = 1.62), (*p* = 0.024). This finding suggests that younger people had a more positive perception of the quality of the “responsiveness” dimension in the health organizations for which they work.

#### 3.4.3. The Specialty Factor

Based on the analysis with ANOVA test (or the Brown–Forsythe when Levene’s test for homogeneity of variance was statistically significant) in order to assess the impact of “Specialty Factor” on the internal quality dimensions, it is shown that the test was statistically significant only for the “Tangibles” dimension (B-FTAN = 8.188, *p* < 0.001, FREL = 1.211, *p* = 0.305, FRES = 2.492, *p* = 0.60, B-FASR = 1.125, *p* = 0.339, B-FEMP = 1.675, *p* = 0.186) (Table 3). The subsequent post hoc analysis based on the Games–Howell test (since the Levene’s test for homogeneity of variances was statistically significant, Levene’s (3, 437) = 2.763, *p* = 0.042) identified statistically significant differences for the pairs: (a) Doctor (mean = 4.81, standard deviation = 1.43) vs. Administrative staff (mean = 3.96, standard deviation = 1.54) (*p* = 0.001) and (b) Nurse (mean = 4.72, standard deviation = 1.28) vs. Administrative staff (mean = 3.96, standard deviation = 1.54) (*p* < 0.001). It could be seen that administrative staff had a lower mean value in both cases. This finding suggests that, when compared to doctors and nurses, administrative staff had a lower perception of the “tangibles” dimension’s quality.

#### 3.4.4. The Education Level Factor

Regarding the “Education Level” factor, the statistical analysis with the one-way ANOVA or the Brown–Forsythe test did not show statistically significant differences for any of the internal quality dimensions (FTAN = 0.795, *p* = 0.554, FREL = 0.961, *p* = 0.441, FRES = 1.162, *p* = 0.327, B-FASR = 1.679, *p* = 0.147, FEMP = 1.009, *p* = 0.412). This finding suggests that education had no effect on employees’ perceptions of internal quality dimensions. However, although not statistically significant, university graduates had higher values for all the internal quality dimensions except for the “responsiveness” one, where higher values were recorded for Ph.D. holders (mean values by dimension and education category: (a) Tangibles: CE: 3.91, SE: 4.36, TE: 4.57, UE: 4.62, M.Sc.: 4.42, Ph.D.: 4.58, (b) Reliability: CE: 3.71, SE: 3.75, TE: 3.75, UE: 3.85, M.Sc.: 3.36, Ph.D.: 3.67, (c) Responsiveness: CE: 3.36, SE: 3.80, TE: 3.97, UE: 4.01, M.Sc.: 3.60, Ph.D.: 4.11, (d) Assurance: CE: 3.39, SE: 3.73, TE: 3.98, UE: 4.00, M.Sc.: 3.43, Ph.D.: 3.96, (e) Empathy: CE: 3.35, SE: 3.54, TE: 3.65, UE: 3.83, M.Sc.: 3.31, Ph.D.: 3.58).

#### 3.4.5. The Professional Experience Factor

Statistical analysis with Spearman’s Rho correlation coefficient showed statistically significant correlations for the dimensions of Reliability, Responsiveness, and Assurance. Specifically, a very weak negative correlation was found between the Years of Experience and (i) the reliability dimension (rho = −0.099, *p* = 0.038), (ii) the responsiveness dimension (rho = −0.117, *p* = 0.014), and (iii) the assurance dimension (rho = −0,130, *p* = 0.006) (Table 8). According to these findings, there was an indication that employees with more previous working experience had a worse perception of the quality of Reliability, Responsiveness, and Assurance dimension.

### 3.5. Impact of Job Satisfaction Subscales on the Internal Quality Dimensions

Based on the statistical analysis with Spearman’s Rho correlation coefficient in order to assess for possible correlations between the Job Satisfaction subscales and the internal quality subscales, it was revealed that (Table 8):

(a) There was a positive and moderate correlation between the JSS1 subscale and all the quality dimensions except for the “tangibles” one, which was also positive but weak. According to this finding, employees who were more satisfied with their earnings had a more positive perception for all the quality dimensions.
(b) There was a positive and moderate correlation between the JSS2 subscale and all the quality dimensions. This finding implies that employees who were more satisfied with the opportunities to develop capabilities and initiatives had a more favorable perception towards all the quality dimensions.
(c) There was a positive and moderate correlation between the JSS3 subscale and all the quality dimensions. This finding suggests that employees who were more satisfied with their operating working conditions had a more favorable perception towards all the quality dimensions.
(d) A positive and moderate correlation had been identified between the JSS4 subscale and all the quality dimensions. As a result, employees who were more satisfied with the recognition and rewards for their work had a more positive perception towards all the quality dimensions.
(e) A positive and weak correlation had been identified between the JSS5 subscale and all the quality dimensions. As a result, employees who were more satisfied with their colleagues and other hospital employees had a more positive perception towards all the quality dimensions.
(f) There was a positive and moderate correlation between the JSS6 subscale and all the quality dimensions except for the “tangibles” one, which was also positive but weak. This result shows that employees who were more satisfied with their profession as a whole had a more favorable perception towards all the quality dimensions.

## 4. Discussion

The importance of human resources in an organization’s successful and effective management cannot be understated. In the last decades, the concept of internal quality service has emerged as a vital approach to services’ management. Additionally, job satisfaction is considered to be a very important aspect for the sustainability and development of health care systems and is regarded as an indirect indicator of services’ quality. Therefore, human resources’ job satisfaction is necessary for the efficient operation of the organizations [62,63]. The relationship between internal quality and job satisfaction is a crucial research subject as this relationship has been recognized as determining the services’ external quality and efficiency [64].

This study with 441 participants, health professionals, from six hospitals in the region of Attica, tried to assess the hospitals’ internal quality and job satisfaction. The Greek version of the SERVQUAL questionnaire and six questions from the JSS questionnaire were utilized as the research tools of the survey [54,55].

Overall, the findings of this survey showed that the employees who participated in the study evaluated the internal quality of services in the hospitals where they work as non-satisfactory with the only exception the tangibles dimension. This finding is in line with the study of Gogos et al. [21] which was carried out in Greece using the same research instrument. However, Gogos et al.’s study disagreed with the findings of other occupational health surveys related to internal quality in the hospital setting, which revealed a positive perception for all the five dimensions of internal quality [65,66].

As previously stated, health care professionals cannot provide excellent service to their patients unless they receive excellent service from the other employees with whom they cooperate to provide the service. In this study, the internal quality of services was evaluated as low. Studies conducted in the country over the last few years to research and analyze patients’ perspectives on external quality found that hospitals’ external quality is also low [67,68,69]. The quality of services delivered to organizational personnel frequently determines how well the external consumers are served [64,70]. If employees endure poor quality of service, then it is likely that service will suffer as a result [3].

Regarding the tangibles quality dimension of the instrument, the participants seemed to have a very favorable impression concerning employees’ physical appearance, the equipment in the departments as well as the attitude and the professionalism of the administrative employees. This finding was in agreement with other relevant studies in which health professionals rated the level of equipment from medium to high [21,55].

Referring to empathy, responsiveness, assurance, and reliability dimensions, the employees’ rated them lower than the neutral value (neutral value = 4). The dimension of reliability was formed through managers’ prompt/timely fulfillment of the promises they made to employees, their real interest in solving the problems that arise in the hospital setting, the provision of reliable information to the professionals, and the maintenance of health data and medical records. The responsiveness dimension referred to the managers’ willingness to offer assistance, immediate response for access to important and critical information, and direct unhindered communication between managers and professionals. The dimension of assurance emphasized managers’ behaviors that built trust for employees. These behaviors were designated by courtesy, consistency, honesty, and practical support so that the employees could perform their duties properly and effectively. Finally, empathy focuses on the knowledge and understanding of the needs of professionals, the priority of meeting these needs, and the ability of the health professionals to seek support and advice from managers during those hours and moments when they are most needed [46,55].

According to this study, health employees did not believe that hospital managers gave them individual attention, nor did they understand the needs and requirements of the department in which each employee works. Furthermore, the employees who participated in this research did not believe that managers are capable of performing the promised service reliably and accurately, and they did not believe that managers inspire confidence.

Concerning job satisfaction, which is a complex and multidimensional concept, the results of this survey showed that the overall employee satisfaction level was very low, mainly because of the higher dissatisfaction appearing in the criteria of earnings and working conditions. Working conditions, in particular, received the lowest score. This can be explained by the fact that the survey took place during the period when hospitals and health care employees were dealing with the pandemic crisis’s (COVID-19) consequences. During this period, hospitals’ staff worked longer hours than usual, with no days off and additionally they were emotionally charged due to the daily deaths of patients.

Referring to the earnings dimension, the results were similar to national and international studies [33,71,72,73,74,75,76,77,78,79]. In the case of Greece, the country’s economic crisis, which has lasted more than ten years, may have contributed to the outcome. Due to the crisis, job dissatisfaction has escalated since the number of employees has decreased, wages have been drastically dropped, and the thirteenth monthly wage has been abolished [63].

On the subject of their interaction with their colleagues and other hospital employees, it appears that the employees who participated in this research were moderately satisfied with their interaction, with practicing their profession as a whole, and with people acknowledging their efforts at work. This finding was similar to the finding of other studies in the health care sector [33,79,80].

As regards the satisfaction with the opportunities and possibilities for improving their skills, the health care professionals of the current study were not satisfied, which is consistent with the findings of other studies [79,81,82,83]. Several prior study efforts in this area have emphasized the importance of educational training programs for enhancing hospital employees [84].

Concerning the relation between internal quality and job satisfaction, the results of the study indicated that it was a strong and positive relation. Employees who were more satisfied with their earnings, with the opportunities to develop capabilities and initiatives, with their operating working conditions, with the recognition of their contribution to their work, with their colleagues and other hospital employees, and with their profession as a whole had a more favorable perception towards all the quality dimensions. The results are in line with other studies [2,24,43,64,85,86,87] which concluded that there is a positive relation between job satisfaction, organizational commitment, organizational effectiveness, growth, and development. The opposite leads employees to poor mental health and lack of physical and mental well-being, resulting in non-positive emotional response to their work [79].

Employee satisfaction is a significant determinant of service quality [29]. Employees who are satisfied are more devoted to continuous development and service quality [30], are more attentive, have higher job morale, perform more efficiently and effectively [32,33] and, as a result, provide excellent service [31]. Health care employees are in close contact with the customer–patient. Therefore, establishing consistent service performance is the most challenging stage in enhancing health care service quality [31].

Gender and educational level factors do not seem to affect employees’ perceptions regarding the internal quality of the hospital where they work. In terms of gender, this finding contradicts the literature, where surveys [42,88] showed that men were less satisfied with their jobs than women, whereas Bouranta et al. found the opposite [24].

Concerning age, the results indicated that younger employees have a more positive perception of the quality of the responsiveness dimension in the health organizations where they work. This result was in contrast with the findings of a study where older employees with more years of seniority ranked higher in scale [18].

In terms of profession, administrative staff had a lower perception of the quality of tangibles dimension than doctors and nurses. This finding was opposed to Breslau et al.’s study [89] which concluded that there was no significant difference between professions.

Concerning years of experience, the results indicated that employees with more previous working experience had a worse perception of the quality of reliability, responsiveness, and assurance dimensions, which is consistent with the findings of another study [24]. One possible explanation is that as a person grows older and acquires more expertise, his demands of his profession expand and therefore, the job satisfaction decreases.

### Limitations of Study

The study had some limitations. Firstly, it was carried out in the midst of a pandemic crisis (COVID-19), a crisis with disastrous implications for the health of society. Secondly, it took place during a period when the country and its public health system were still struggling to recover from the multiyear global financial crisis of the past decade. In the midst of these two situations, the health system absorbed a lot of vibrations. This period of time has led health professionals to work harder, resulting in physical fatigue and mental exhaustion. Therefore, the low rating of the internal quality and the satisfaction of health care professionals may have been influenced by the above-mentioned two factors. Further, convenience sampling was utilized, which added a systematic selection error and prevented the results from being generalized. In addition, because the survey’s findings were limited to six hospitals in Attica, the results may only be applied to specific institutions and may not accurately reflect health professionals’ views of the region’s public health services. Further research could be performed in different-sized hospitals, in provincial hospitals, or private hospitals to see if there are differences in the hospitals’ internal quality. Future qualitative research that will thoroughly examine the role of internal quality in determining job satisfaction would result in more accurate results.

## 5. Conclusions

The way health care professionals perceive the provided service’s quality and the level of health care employees’ job satisfaction are important tools for implementing the principles of continuous quality improvement in the health sector and could be systematically measured.

This study made an attempt, firstly, to evaluate employees’ perceptions regarding the internal quality of offered public health services and secondly to investigate whether there is a relation between internal quality and health care professionals’ job satisfaction. According to the study’s findings, the level of internal quality service was found to be low. The results also revealed a positive correlation between the job satisfaction subscales and the quality dimensions. Regarding demographic characteristics and their effect on employees’ perceptions of internal quality dimensions, the study found that the gender and the educational factor had no effect while younger employees have a more positive perception of the quality of responsiveness dimension in the health organizations where they work. In terms of profession, administrative staff had a lower perception of the quality of tangibles dimension than doctors and nurses. Concerning years of experience, the results indicated that employees with more previous working experience had a worse perception of the quality of reliability, responsiveness, and assurance dimensions.

As the health care professionals stated, the hospital managers are not able to recognize the aspects that influence the well-being and contentment of health professionals in order to achieve the best possible results from hospital employees. In addition, they are not focused on their ability to deliver the promised service consistently and precisely, they do not have the knowledge and the ability to motivate health professionals, they do not pay special attention to employees, and are not willing to assist employees in providing direct service.

## Figures and Tables

**Table 1:** Results of the Cronbach’s alpha coefficient.

SERVQUAL	Cronbach’s Alpha	N of Items
Tan	0.795	4
Rel	0.922	5
Res	0.886	4
Asr	0.937	4
Emp	0.914	5
SERVQUAL Total	0.964	22
JSS Total	0.872	6

Tan: Tangibles, Rel: Reliability, Res: Responsiveness, Asr: Assurance, Emp: Empathy, JSS: Job Satisfaction Survey.

**Table 2:** Normality tests for Quality Dimensions and “Years of Experience”.

Dimension	Kolmogorov–Smirnov ^a^		
	Statistic	df	Sig.
Tan	0.078	441	<0.001
Rel	0.059	441	0.001
Res	0.050	441	0.011
Asr	0.058	441	0.001
Emp	0.060	441	0.001
Years of Experience	0.124	440	<0.001

^a^ Lilliefors significance correction. Tan: Tangibles, Rel: Reliability, Res: Responsiveness, Asr: Assurance, Emp: Empathy.

**Table 3:** Demographic characteristics of the respondents and impact evaluation of the demographic characteristics on internal quality dimensions.

Demographic Characteristics		Frequency	Percent	Tangibles	Reliability	Responsiveness	Assurance	Empathy
				Test (df) *p*-Value	Test (df) *p*-Value	Test (df) *p*-Value	Test (df) *p*-Value	Test (df) *p*-Value
Gender	Male	121	17.4	*t* * = 0.157 (439) *p* = 0.875	*t* * = 0.657 (439) *p* = 0.881	*t* * = −0.204 (439) *p* = 0.511	*t* * = −0.022 (439) *p* = 0.838	*t* * =−0.09 (439) *p* = 0.928
	Female	320	72.6					
Age Groups	≤30	50	11.3	B-F ** = 0.565 (3, 437) *p* = 0.638	F *** = 2.484 (3, 437) *p* = 0.06	F *** = 2.995 (3, 437) *p* = 0.031	F *** = 2.186 (3, 437) *p* = 0.089	F *** = 0.878 (3, 437) *p* = 0.453
	31–40	151	34.2					
	41–50	144	32.7					
	≥51	96	21.8					
Specialty	Nurse	74	16.8	B-F ** = 8.188 (3, 325) *p* < 0.001	F *** = 1.211 (3, 437) *p* = 0.305	F *** = 2.492 (3, 437) *p* = 0,60	B-F ** = 1.125 (3, 375) *p* = 0.339	B-F *** = 1.675 (3, 371) *p* = 0.186
	Doctor	182	41.3					
	Administrative staff	106	24.0					
	Other	79	17.9					
Education Level	CE ^	11	2.5	F *** = 0.795 (5, 435) *p* = 0.554	F *** = 0.961 (5, 435) *p* = 0.441	F *** = 1.162 (5, 435) *p* = 0.327	B-F ** = 1.679 (5, 93) *p* = 0.147	F *** = 1.009 (5, 435) *p* = 0.412
	SE ^^	125	28.3					
	TE ^^^	141	32.0					
	UE ^^^^	63	14.3					
	M.Sc.	80	18.1					
	Ph.D.	21	4.81					
Previous Experience				Mean = 15.88	Median = 14.00	Std. deviation = 10.87	Minimum = 1.00	Maximum = 42.00

^ Compulsory Education, ^^ Secondary Education, ^^^ Technological Education, ^^^^ University Education, * independent samples *t*-test test, ** Brown–Forsythe test, *** ANOVA test.

**Table 4:** The five (5) questions with the highest score.

SERVQUAL Dimensions	Question	Mean Value	Std. Deviation	Minimum	Maximum
Tangibles	The hospital staff should be well dressed and appear neat.	5.75	1.45	1	7
Tangibles	The equipment of my department is suitable for the type of provided services.	4.35	1.84	1	7
Assurance	The hospital managers are always kind and friendly to me.	4.23	1.76	1	7
Empathy	The hospital managers work during my department’s convenient hours.	4.18	1.86	1	7
Tangibles	My department has up-to-date equipment.	4.08	1.83	1	7

**Table 5:** The five (5) questions with the lowest score.

SERVQUAL Dimensions	Question	Mean Value	Std. Deviation	Minimum	Maximum
Empathy	The hospital managers always give me individual attention.	3.28	1.80	1	7
Empathy	The hospital managers always understand the needs of my department.	3.35	1.69	1	7
Empathy	The hospital managers are always concerned with my department’s best interest.	3.48	1.76	1	7
Responsiveness	The hospital managers always provide their services when they promise to do so.	3.49	1.64	1	7
Assurance	The hospital managers’ behavior inspires complete confidence in me.	3.55	1.68	1	7

**Table 6:** An overview of selected job satisfaction subscales.

Subscales in Job Satisfaction	Mean Values	Std. Deviation	Minimum	Maximum
Salary satisfaction	3.06	1.32	1	6
Satisfaction related to opportunities to develop capabilities and initiatives	3.10	1.38	1	6
Satisfaction related to working conditions	2.99	1.45	1	6
Satisfaction related to contingent rewards	3.14	1.54	1	6
Satisfaction related to colleagues and other hospital employees	4.55	1.18	1	6
Overall job satisfaction	3.88	1.32	1	6

**Table 7:** One-sample *t*-test results for comparing SERVQUAL dimensions to the neutral value.

Dimension	N	Mean	*t*-Test	*p*-Value
Tangibles	441	4.47	7.03	<0.001
Reliability	441	3.69	−4.38	<0.001
Responsiveness	441	3.85	−2.09	0.037
Assurance	441	3.80	−2.70	0.007
Empathy	441	3.58	−5.81	<0.001

**Table 8:** Spearman’s Rho correlation coefficient results (a) among years of experience and quality dimensions and (b) job satisfaction subscales and quality dimensions.

Dimensions	Spearman’s Rho	Tangibles	Reliability	Responsiveness	Assurance	Empathy
YofE	Correlation Coefficient Sig (2-tailed)	−0.009 <0.847	−0.099 * 0.038	−0.117 * 0.014	−0.130 ** 0.006	−0.089 <0.063
JSS1	Correlation Coefficient Sig (2-tailed)	0.348 ** <0.001	0.429 ** <0.001	0.406 ** <0.001	0.429 ** <0.001	0.454 ** <0.001
JSS2	Correlation Coefficient Sig (2-tailed)	0.436 ** <0.001	0.538 ** <0.001	0.531 ** <0.001	0.576 ** <0.001	0.556 ** <0.001
JSS3	Correlation Coefficient Sig (2-tailed)	0.547 ** <0.001	0.499 ** <0.001	0.504 ** <0.001	0.516 ** <0.001	0.527 ** <0.001
JSS4	Correlation Coefficient Sig (2-tailed)	0.406 ** <0.001	0.458 ** <0.001	0.490 ** <0.001	0.515 ** <0.001	0.539 ** <0.001
JSS5	Correlation Coefficient Sig (2-tailed)	0.304 ** <0.001	0.302 ** <0.001	0.319 ** <0.001	0.291 ** <0.001	0.296 ** <0.001
JSS6	Correlation Coefficient Sig (2-tailed)	0.395 ** <0.001	0.448 ** <0.001	0.439 ** <0.001	0.437 ** <0.001	0.436 ** <0.001

YoE: Years of Experience, JSS1: Salary satisfaction, JSS2: Satisfaction from opportunities to develop capabilities and initiatives, JSS3: Satisfaction from the operating working conditions, JSS4: Satisfaction from contingent rewards, JSS5: Satisfaction from colleagues and other hospital employees, JSS6: Overall job satisfaction. * Significant at 0.05. ** Significant at 0.01.

## Data Availability

Restrictions apply to the availability of these data. Data were collected from Postgraduate Program “Health and Social Care Management” of the Department of Business Administration of the University of West Attica and are available from Goula A. with the permission of MSc Program.
